# Overexpression of *MzASMT 1*, a Gene From *Malus zumi* Mats, Enhances Salt Tolerance in Transgenic Tobacco

**DOI:** 10.3389/fpls.2020.561903

**Published:** 2020-10-26

**Authors:** Weibing Zhuang, Tianyu Liu, Xiaochun Shu, Hongxue Wang, Zhong Wang, Tao Wang, Fengjiao Zhang, Shenchun Qu

**Affiliations:** ^1^College of Horticulture, Nanjing Agricultural University, Nanjing, China; ^2^Institute of Botany, Jiangsu Province and Chinese Academy of Sciences (Nanjing Botanical Garden, Memorial Sun Yat-sen), Nanjing, China

**Keywords:** melatonin, *MzASMT 1*, salt tolerance, ROS, antioxidant enzyme system

## Abstract

Melatonin, widely found in various plants as a new antioxidant, could protect plants from various biotic and/or abiotic stresses, including salt stress. *MzASMT 1* (KJ123721), a gene from *Malus zumi* Mats, is a key enzyme required for melatonin synthesis. However, whether the overexpression of *MzASMT 1* could regulate the synthesis of melatonin and improve the salt tolerance in tobacco remains unknown. In this study, the overexpression of *MzASMT 1* in tobacco increased the melatonin content, and the transgenic lines owned higher salt tolerance capacity. The transgenic lines overexpressing *MzASMT 1* exhibited lower degree of leaf wilting; much more fresh weight; higher plant height; longer root; higher relative water content (RWC) of leaves, stem, and root; and higher chlorophyll content and Fv/Fm, which makes transgenic lines better adapt to salt stress. The transgenic lines also had higher accumulation of proline, lower accumulation of malondialdehyde (MDA), and improved antioxidant systems, which protected plants from cell damage and oxidative stress due to excess reactive oxygen species (ROS) accumulation under salt treatment. The transcription of salt response genes was much more highly activated in transgenic lines than in wild type under salt stress. The above results contributed to the understanding of functions for *MzASMT 1* in tobacco under salt stress and provided a new choice for the application of *MzASMT 1* in improving plant salt tolerance.

## Introduction

Salinity is one of the common adversities plants endure, which limits the geographical distribution of plants and reduces crop productivity and quality. Up to now, more than 1/3 of the world’s irrigated lands are subjected to different levels of salinization, leading to serious agricultural production loss (Arzani, 2008; Fahad et al., 2015). Due to climate change in the world (such as increase of temperature and decrease of average annual rainfall), salinization continues to occur worldwide. Salt stress has adverse effects on plants through the regulation of photosynthesis, specific ion toxicity, osmotic stress, reactive oxygen species (ROS), and so on (Zhu, 2001; Hasanuzzaman et al., 2014; Abbasi et al., 2016; Liang et al., 2018). The cells and organs of plants could generate excessive ROS when plants are subjected to high salinity stress, and excessive ROS lead to worse growth including programmed cell death and eventually plant death (Dat et al., 2003; Krishnamurthy and Rathinasabapathi, 2013). Therefore, it is of vital importance to explore the molecular mechanism of salinity tolerance in order to regulate the growth and development of plants in salinized soil.

Melatonin, first reported in plants in 1995, has various physiological functions in plants, including formation of the rhizome; promotion of explant growth; and regulation of circadian rhythms, photosynthesis, flowering, and seed germination (Murch and Saxena, 2002; Hernández-Ruiz et al., 2004, 2005; Arnao and Hernández-Ruiz, 2006, 2007, 2009a, 2018; Tan et al., 2012; Reiter et al., 2015). Melatonin could scavenge ROS and reactive nitrogen species (RNS) in the cells of plants, which can protect plants from various biotic/abiotic stresses as a scavenger (Tan et al., 2012; Yu et al., 2018). As melatonin is an amphiphilic molecule, it can remove the excess ROS in all cellular compartments including the cytoplasm, membrane, nucleus, and mitochondria, which is different from the classic antioxidants, such as the enzymes of peroxidase (POD), superoxide dismutase (SOD), and catalase (CAT) (Venegas et al., 2012; Reiter et al., 2013). In addition, the metabolites of melatonin can also remove the ROS of cells, which enhances the anti-oxidative capacity of melatonin (Galano et al., 2013). The ability of neutralizing ROS for both melatonin and its metabolites is referred as the scavenging cascade reaction (Rosen et al., 2006).

Many researches indicated that exogenous melatonin treatment could relieve plants from various biotic and abiotic stresses (Gu et al., 2017; Yu et al., 2018; Zhao et al., 2018; Aghdam et al., 2019; Zhan et al., 2019), and more and more researchers focused on the functions of endogenous melatonin in plants through genetic engineering. The biosynthesis of melatonin in plants is as follows: firstly, tryptamine is formed through the decarboxylation of tryptophan; secondly, serotonin is formed through the hydroxylation of tryptamine; thirdly, *N*-acetyl serotonin is acquired by serotonin N-acetyltransferase (SNAT) from serotonin, and then melatonin is obtained through two kinds of enzymes: acetyl serotonin methyl transferase (ASMT) or caffeic acid *O*-methyltransferase (COMT) from *N*-acetyl serotonin (Fujiwara et al., 2010; Byeon et al., 2014a; Lee et al., 2014; Wang et al., 2014). However, Byeon et al. (2014b) observed that the catalytic activity of COMT was more than 700-fold that of ASMT during melatonin synthesis in rice, and the overexpression of *COMT* could increase the concentration of melatonin and enhance the salt stress tolerance in various plants. As the catalytic activity of ASMT is not high in plants, the *N*-acetylserotonin methyltransferase (*ASMT*) is considered the rate-limiting step in the melatonin synthesis process (Park et al., 2013; Byeon et al., 2014a). Recently, many researchers focus on the functions of *COMT* in various plants under salt treatment (Liu D.D. et al., 2019; Sun et al., 2019; Zhang et al., 2019). However, few studies reported the functions of *ASMT* in plants under various stress, especially under salt stress. *MzASMT1*, a gene from a salt-tolerant apple species *Malus zumi*, overexpression could enhance drought tolerance in transgenic *Arabidopsis thaliana* (Zuo et al., 2014). Compared with the function analysis of *COMT* under salt stress, the functions of *ASMT* in plants under salt treatment need to be further explored.

In present study, *MzASMT 1* was synthesized, and the functions of which in tobacco under salt stress have been investigated. The transgenic tobacco overexpressing *MzASMT 1* had better growth status compared with wild type (WT) under salt stress condition. The photosynthetic efficiency, proline and malondialdehyde (MDA) content, H_2_O_2_ and O_2_^⋅–^ content, activities of antioxidant enzymes, and the expression level of stress-related genes in transgenic tobacco under salt stress were evaluated. The results could enhance our understanding of *MzASMT 1* functions in plants under salt stress in theory and facilitate practical applications to improve salt tolerance of tobacco and other plants.

## Materials and Methods

### Plant Materials

Tobacco (*Nicotiana tabacum* L.) cultivar K326 was used as experimental material in this study. All plant materials were provided by the College of Horticulture, Nanjing Agricultural University, Nanjing, China.

For tobacco explant preparation, the WT tobacco seeds were surface sterilized using 75% (*v*/*v*) ethanol for 30 s and then immersed for 10 min in 12% (*v*/*v*) NaClO solution. After that, the sterilized seeds were washed five times with sterilized water. The sterilized seeds were sowed on horizontal plates containing Murashige and Skoog (MS) medium at pH 5.8 ± 0.5, and the plates were placed under 25 ± 2°C with a 16-h light and 8-h dark photoperiod cycle for 2 weeks. The strong and healthy tobacco seedlings were transferred into tissue culture bottles containing MS medium at 1 month for genetic transformation.

### Vector Construction

The cDNA fragment of *MzASMT 1* gene including restriction enzyme sites *Xba*I and PastI was synthesized by GenScript (Nanjing, China) and then inserted into pCAMBIA2300 vector with *Xba*I and PastI restriction enzyme sites to construct the recombined vector pCAMBIA2300-*MzASMT 1*. The sequence of cDNA fragment for *MzASMT 1* gene was obtained from NCBI^1^, and the accession number is KJ123721. The constitutive expression system included Cauliflower mosaic virus (CaMV) 35S promoter, nopaline synthase (NOS) terminator system, β-glucuronidase gene (GUS), and kanamycin-resistant gene in the recombined vector pCAMBIA2300-*MzASMT 1*.

### Tobacco Transformation and Identification

The pCAMBIA2300-*MzASMT 1* was transformed into *Agrobacterium tumefaciens* EHA105. Transgenic tobacco plants were obtained using the *Agrobacterium-*mediated method as previously described (Guo et al., 2012). Transgenic tobacco lines were selected on MS medium containing 50 mg l^–1^ kanamycin (Kan) and 100 mg l^–1^ Timentin (Tim). The candidate transgenic T_0_ lines were screened from regenerated Kan-resistant plants, and the positive transgenic T_0_ plants were further verified by GUS staining and polymerase chain reaction (PCR) detection. WT plants were used as controls.

### Salt Stress Treatment

For salt treatment, three lines (OE-1, OE-2, and OE-3) were selected from transgenic lines overexpressing *MzASMT 1*, and WT were used as control. Each line contained 10 plants to conduct the salt treatment, and three independent experiments were performed for each parameter measurement. Firstly, tissue culture seedlings were grown for 20 days in the tissue culture room. Secondly, they were placed in a growth chamber for adaption to grow better. After that, they were transplanted into soil in plastic pots (30 cm × 25 cm × 22 cm) in the greenhouse. They were watered continuously every 3 days for 10 days to maintain healthy growth. The tobacco seedlings were irrigated with 200 mM NaCl solutions every 2 days for salt treatment and with water every 2 days as control. After 14 days of the treatment, the plants were carefully photographed. Seven days after 14 days of salt treatment, the survival rate of transgenic lines and WT was evaluated. In addition, the tissues of leaves and roots were also collected and then frozen in liquid nitrogen for further analysis, such as the evaluation of physiological index and gene expression analyses. Three independent experiments were performed for each parameter measurement.

### Determination of Plant Growth Index and Physiological Trait

After 14 days of salt treatment, plant height, root length, and fresh weight were measured with a Vernier caliper and scale. The relative water content (RWC) of the leaves, stem, and root was calculated according to protocols described by Virginia et al. (2012). Total chlorophyll contents were evaluated with a method previously described (Gao and Peng, 2006). The maximum quantum efficiency of photosystem II photochemistry, Fv/Fm, was determined after dark adaptation for 30 min with an Open FluorCam 701MF imaging fluorometer (Photon Systems Instruments, Brno, Czechia) as described by Baba et al. (2012).

To explore the effects of physiological changes caused by the overexpression of *MzASMT 1* in transgenic tobacco lines under salt treatment, similar leaves were collected from WT and transgenic plants after 14 days of salt treatment. The content of proline and MDA and the activities of POD, SOD, and CAT were determined according to a previous method (Zong et al., 2009; Duan et al., 2012; Zhao et al., 2013; Ryu et al., 2014). The histochemical assay of hydrogen peroxide (H_2_O_2_) and superoxide anion (O_2_^⋅–^) was carried out with diaminobenzidine (DAB) and nitrotetrazolium blue chloride (NBT) as chromogenic substrates, respectively (Kumar et al., 2013), and the content of H_2_O_2_ and O_2_^⋅–^ was determined with the method described by Zhou et al. (2014).

### Determination of Melatonin by Enzyme-Linked Immunosorbent Assay

Wild type and transgenic tobacco lines with uniform growth potential were weighed, and 0.1 g of fresh leaves in both WT and transgenic tobacco lines were used to determine the melatonin content. Melatonin content in the leaves was measured using an enzyme-linked immunosorbent assay (Shanghai Enzyme Biotechnology, Shanghai, China). The standard, blank, and sample wells were assayed individually, and the absorbance at 450 nm was measured. The standard curve was generated after measuring the standard products, and the transgenic tobacco lines and WT were assayed individually.

### RNA Extraction, cDNA Synthesis, and Quantitative Real-Time PCR Analysis

Total RNA was extracted according to manufacturer instructions, and the extracted RNA was then reverse transcribed using a PrimeScript^TM^ 1st Strand cDNA Synthesis Kit according to the kit instructions. Quantitative real-time PCR (qRT-PCR) was performed using TB Green^TM^ Premix Ex Taq^TM^ II (Tli RNaseH Plus) (Takara). Each 20-μl quantitative real-time PCR contained 10 μl of TB Green^TM^ PCR master mix, 0.2 mM of each primer, and 10 ng of cDNA with the following PCR program: 95°C for 5 min, followed by 40 cycles of 95°C for 15 s, and 62°C for 1 min in an ABI 7300 Real-Time PCR System (Applied Biosystems, Foster City, CA, United States). *NtTubulin* (N181029A17) was used as a house-keeping gene to investigate gene expression in transgenic tobacco lines overexpressing *MzASMT 1* and WT. All gene-specific primers were designed with Primer 5 software and are listed in Supplementary Table S1. The relative abundance of the genes was determined with 2^−ΔΔCT^ method (Livak and Schmittgen, 2001). Each qRT-PCR analysis was repeated three times.

### Statistical Analysis

The experiments were repeated three times with three biological replicates. All data were expressed as mean ± standard error (SE). Differences among means of the various treatments were determined by the least significant difference test. Significance analysis was performed using SPSS 17.0 software. Means were considered to be significantly different when *P* ≤ 0.05. Significance analysis was performed using SPSS 17.0 software. DNAMAN, Microsoft Excel, and GraphPad Prism 5.0 software were used for data analysis and charting.

## Results

### Generation and Identification of Transgenic Tobacco Lines

**FIGURE 1:**
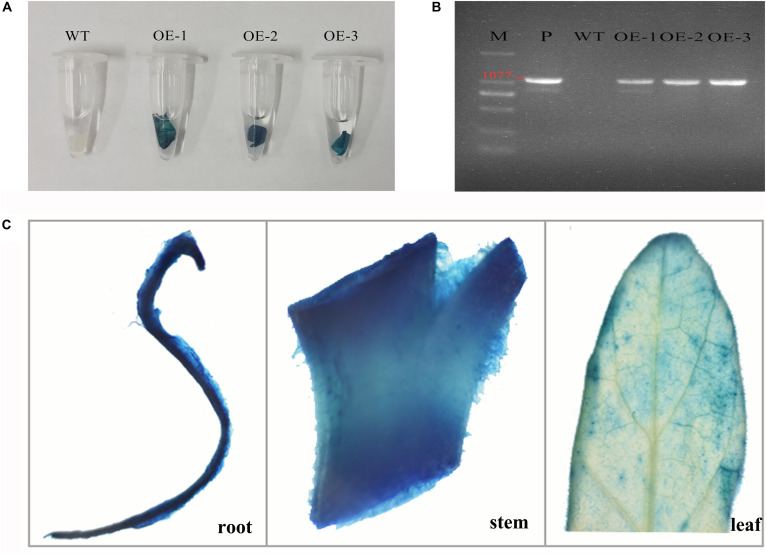
GUS staining **(A)** and genomic detection **(B)** of transgenic tobacco lines overexpressing *MzASMT 1* (M, marker; P, positive control by using pCAMBIA2300-*MzASMT 1* plasmid DNA as PCR template; WT, wild type; OE-1, transgenic line OE-1; OE-2, transgenic line OE-2; OE-3, transgenic line OE-3). The GUS staining of different tissues (roots, stems, and leaves) in transgenic tobacco plants **(C)**.

To characterize the functions of *MzASMT 1* gene in plants under salt stress, several transgenic tobacco lines overexpressing *MzASMT 1* were generated. The transgenic tobacco lines were further verified by GUS staining and genomic PCR. The results indicated that transgenic lines OE-1, OE-2, and OE-3 had a positive GUS staining compared with WT (Figure 1A), and the target fragment of *MzASMT 1* with special primers (1077 bp) was amplified from transgenic lines OE-1, OE-2, and OE-3 by genomic PCR (Figure 1B). GUS histochemical assays revealed that the staining in stems and roots in transgenic tobacco plants is much more intense than that in leaves (Figure 1C). Therefore, transgenic lines OE-1, OE-2, and OE-3 were further used to explore the functions of *MzASMT 1* gene in tobacco under salt stress.

### *MzASMT 1* Overexpression Contributes to Salt Tolerance in Transgenic Tobacco Under Salt Stress

**FIGURE 2:**
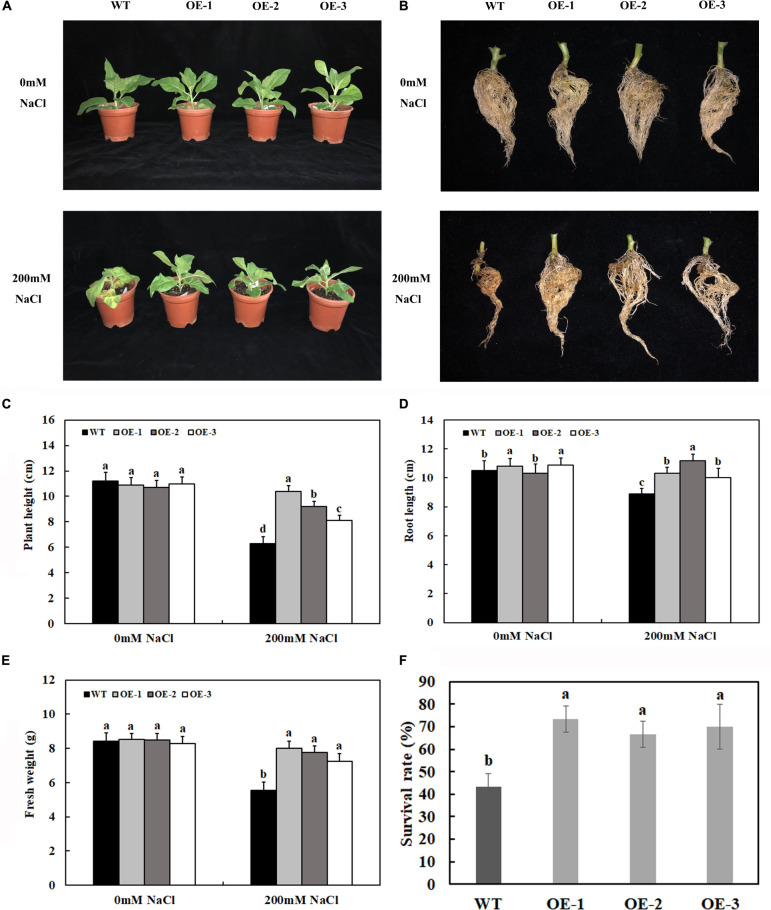
Growth status of transgenic lines and WT after salt treatment and water treatment **(A)**. Root growth **(B)**, plant height **(C)**, root length **(D)**, fresh weight **(E)**, and survival rate **(F)** of transgenic lines and WT after salt treatment and water treatment. Data are means ± SE of three biological replicates and means followed by different letters are significantly different (*P* < 0.05) (WT, wild type; OE-1, transgenic line OE-1; OE-2, transgenic line OE-2; OE-3, transgenic line OE-3).

Under control conditions, there were no significant differences in plant growth between the transgenic plants overexpressing *MzASMT 1* and WT, such as plant height and fresh weight (Figures 2A,C,E). After 7 days of salt treatment, there were no significant differences between the phenotypes of transgenic lines and WT. However, after 14 days of salt treatment, the transgenic lines exhibited lower degrees of leaf wilting than WT (Figure 2A). To better understand the functions for *MzASMT 1* in tobacco under salt stress, the physiological data of transgenic lines and WT after 7 days of salt treatment were also evaluated. The plant height and fresh weight of transgenic lines were much higher than those of WT (Figures 2C,E), and the root of transgenic lines was much longer than that of WT (Figures 2B,D) after 14 days of salt treatment. The survival rate of transgenic lines is significantly higher than that of WT after 1 week of salt stress recovery (Figure 2F). The above results showed that transgenic lines exhibited higher growth potential than WT under salt treatment, which indicated that transgenic tobacco plants overexpressing *MzASMT 1* enhanced their salt tolerance under salt treatment.

**FIGURE 3:**
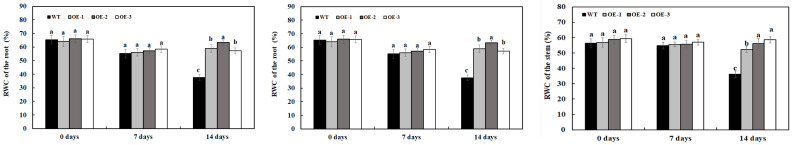
RWC of the root, stem, and leaves in transgenic lines and WT after salt treatment and water treatment. Data are means ± SE of three biological replicates, and means followed by different letters are significantly different (*P* < 0.05) (WT, wild type; OE-1, transgenic line OE-1; OE-2, transgenic line OE-2; OE-3, transgenic line OE-3).

Relative water content is one of the several indexes to measure plant water status and can be used as physiological parameter to indicate salt tolerance of plants. In the present study, the RWC of transgenic plants overexpressing *MzASMT 1* and WT was determined to analyze their salt tolerance under 200-mM NaCl treatment and water treatment. There was no significant difference in RWC in the leaves, stem, and root between transgenic lines overexpressing *MzASMT 1* and WT under water treatment (Figure 3). After 7 days of 200-mM NaCl treatment, there was a slight reduction in the RWC of the leaves, stem, and root in both transgenic lines and WT, and there was no significant difference in RWC in the leaves, stem, and root between transgenic lines overexpressing *MzASMT 1* and WT (Figure 3). After 14 days of 200-mM NaCl treatment, the RWC of the leaves, stem, and root in WT was significantly reduced compared with that in transgenic lines, and transgenic lines had much higher RWC of the leaves, stem, and root compared with that in WT (Figure 3).

**FIGURE 4:**
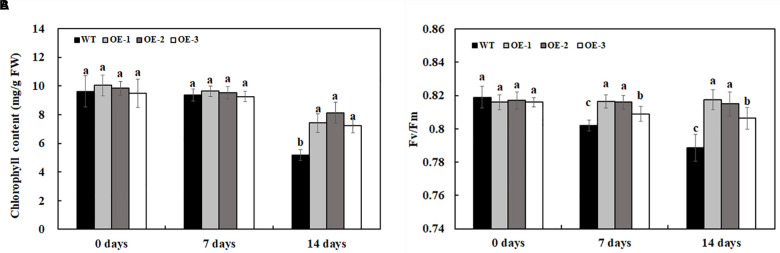
Chlorophyll content **(A)** and Fv/Fm **(B)** in transgenic lines and WT after salt treatment and water treatment. Data are means ± SE of three biological replicates, and means followed by different letters are significantly different (*P* < 0.05) (WT, wild type; OE-1, transgenic line OE-1; OE-2, transgenic line OE-2; OE-3, transgenic line OE-3).

The chlorophyll content and the maximum quantum efficiency of photosystem II photochemistry, Fv/Fm, were quantified to determine the extent of stress in transgenic lines overexpressing *MzASMT 1* and WT. The chlorophyll content had no significant differences between transgenic lines and WT under water treatment. After 7 days of 200-mM NaCl treatment, there was a slight reduction of chlorophyll content for both transgenic lines and WT but there was no significant differences between them (Figure 4A). After 14 days of 200-mM NaCl treatment, the chlorophyll content of WT decreased much more compared with that of transgenic lines, and there were significant differences between the chlorophyll content of transgenic lines and WT (Figure 4A). Fv/Fm, the most widely used parameter, could be used to detect the stress status of plants subjected through a rapid non-destructive method. The higher the Fv/Fm value, the lower the stress status of plants. In the present results, there was no significant difference in Fv/Fm between transgenic lines overexpressing *MzASMT 1* and WT under water treatment. After 7 days of 200-mM NaCl treatment, the Fv/Fm value in WT decreased significantly, while the transgenic lines maintained a higher level (Figure 4B). After 14 days of 200-mM NaCl treatment, the Fv/Fm value in WT decreased much more than that in transgenic lines, and there were significant differences in the Fv/Fm value between transgenic lines and WT (Figure 4B), which indicated that transgenic lines had an enhanced salt tolerance compared with WT.

### *MzASMT 1* Overexpression Enhanced Antioxidant Capacity of Plants Under Salt Stress

**FIGURE 5:**
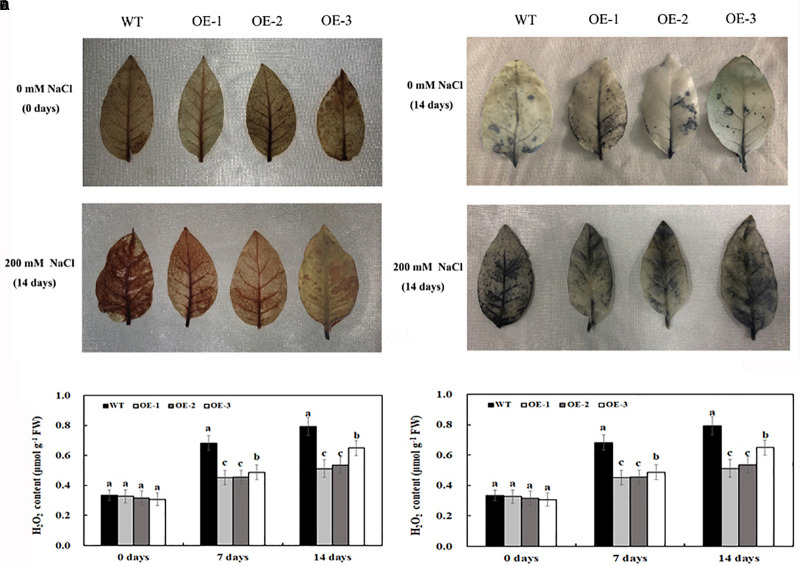
Histochemical staining with DAB for detection of H_2_O_2_ **(A)** and with NBT for detection of O_2_^⋅–^ **(B)** in transgenic lines and WT after salt treatment and water treatment. The content of H_2_O_2_ **(C)** and O_2_^⋅–^ **(D)** in transgenic lines and WT after salt treatment and water treatment. Data are means ± SE of three biological replicates, and means followed by different letters are significantly different (*P* < 0.05) (WT, wild type; OE-1, transgenic line OE-1; OE-2, transgenic line OE-2; OE-3, transgenic line OE-3).

**FIGURE 6:**
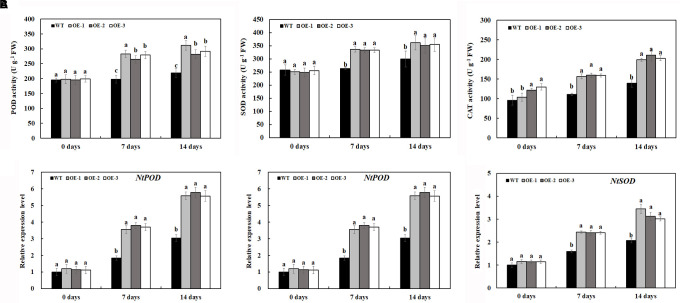
Antioxidant enzyme activities **(A)** and gene expression level **(B)** in transgenic lines and WT after salt treatment and water treatment. Data are means ± SE of three biological replicates, and means followed by different letters are significantly different (*P* < 0.05) (WT, wild type; OE-1, transgenic line OE-1; OE-2, transgenic line OE-2; OE-3, transgenic line OE-3).

Under normal conditions, O_2_^⋅–^ and H_2_O_2_ accumulation was not significantly different between the transgenic lines and WT (Figures 5A–D); however, salt treatment induced the significant accumulation of O_2_^⋅–^ and H_2_O_2_ in both transgenic lines and WT (Figures 5A–D). After 7 days of 200-mM NaCl treatment, the content of O_2_^⋅–^ and H_2_O_2_ was induced quickly, and the content of O_2_^⋅–^ and H_2_O_2_ in WT was much higher than that in transgenic lines. After 14 days of 200-mM NaCl treatment, the content of O_2_^⋅–^ and H_2_O_2_ increased slowly, and the content of O_2_^⋅–^ and H_2_O_2_ in WT was also much higher than that in transgenic lines (Figures 5A–D). Similarly, under normal condition, the activities of POD and SOD had no significant difference between the transgenic lines and WT, which is consistent with the expression level of POD and SOD genes under normal conditions (Figures 6A,B). After 7 days of 200-mM NaCl treatment, the activities of POD, SOD, and CAT for both transgenic lines and WT increased quickly and had a slight increase after 14 days of 200-mM NaCl treatment. However, the activities of POD, SOD, and CAT in transgenic lines are much higher than those in WT (Figure 6A), which was also in agreement with the expression level of POD, SOD, and CAT genes under salt treatment (Figure 6B).

**FIGURE 7:**
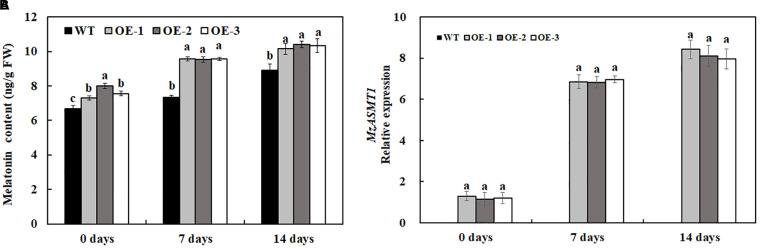
The melatonin content in transgenic tobacco lines and WT after salt treatment and water treatment **(A)**, and the expression level of *MzASMT 1* in transgenic tobacco lines overexpressing *MzASMT1* after salt treatment **(B)**. Data are means ± SE of three biological replicates, and means followed by different letters are significantly different (*P* < 0.05) (WT, wild type; OE-1, transgenic line OE-1; OE-2, transgenic line OE-2; OE-3, transgenic line OE-3).

Melatonin, a naturally potent scavenger in plants, could protect plants from various biotic and abiotic stresses through removal of excess ROS. In our results, the overexpression of *MzASMT 1* in tobacco enhanced their melatonin content, and salt treatment also induced the accumulation of melatonin (Figure 7A). In accordance with the melatonin content, the expression level of *MzASMT 1* in transgenic lines increased significantly after salt treatment (Figure 7B). The higher content of melatonin in transgenic lines could reduce the ROS accumulation due to the salt stress. Therefore, transgenic lines overexpressing *MzASMT 1* had an enhanced salt tolerance compared with WT.

### Overexpression of *MzASMT 1* Increases Proline Accumulation and Decreases MDA Accumulation in Tobacco Plants Under Salt Stress

**FIGURE 8:**
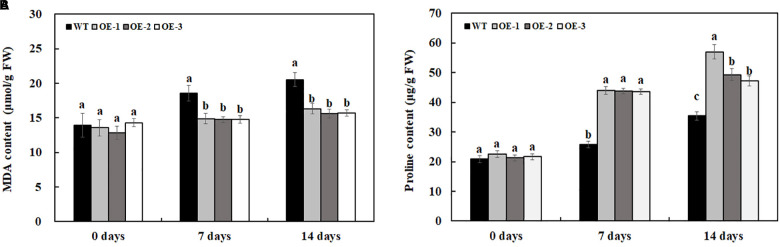
MDA **(A)** and proline **(B)** content in transgenic lines and WT after salt treatment and water treatment. Data are means ± SE of three biological replicates, and means followed by different letters are significantly different (*P* < 0.05) (WT, wild type; OE-1, transgenic line OE-1; OE-2, transgenic line OE-2; OE-3, transgenic line OE-3).

There was no significant difference in proline and MDA content between transgenic lines and WT under water treatment (Figure 8). After 7 days of 200-mM NaCl treatment, the MDA content is much lower in transgenic lines than that in WT, and the MDA content is also much lower in transgenic lines than that in WT after 14 days of 200-mM NaCl treatment (Figure 8A). After 7 days of 200-mM NaCl treatment, the proline content in transgenic lines is much higher than that in WT, and the proline content is also much lower in transgenic lines than that in WT after 14 days of 200-mM NaCl treatment (Figure 8B). The above results indicated that transgenic lines had higher salt tolerance compared with WT.

### Expression Level of Stress-Related Genes in Tobacco Plants Under Salt Stress

In response to environmental stress, plants modulate the expression of a large number of stress response genes, constituting an important molecular basis for the response and adaptation of plants to stresses. To further investigate the gene expression pattern of transgenic tobacco lines overexpressing *MzASMT 1* under salt stress, transcript levels of salt stress-related genes were examined in transgenic lines and WT under normal and salt stress conditions, including genes associated with stress defense (*NtERD10C*, *NtERD10D*, and *NtLEA5*), biosynthesis of proline (*NtP5CS*), and dehydration-responsive element-binding (*DREB*) transcription factor.

**FIGURE 9:**
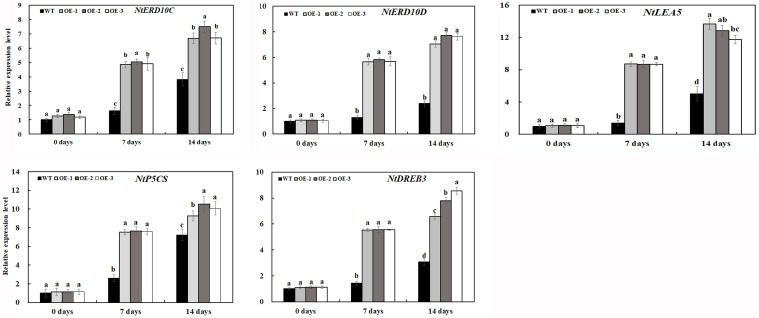
Expression level of salt-related genes in transgenic lines and WT after salt treatment and water treatment. Data are means ± SE of three biological replicates, and means followed by different letters are significantly different (*P* < 0.05) (WT, wild type; OE-1, transgenic line OE-1; OE-2, transgenic line OE-2; OE-3, transgenic line OE-3).

As shown in Figure 9, under normal conditions, the relative expression levels of all eight genes in transgenic lines OE-1, OE-2, and OE-3 were similar with those in WT. The expression levels of genes associated with stress defense in transgenic tobacco lines overexpressing *MzASMT 1* were much higher than those in WT under salt treatment. Although the expression of *NtP5CS* gene in WT increased sharply after 14 days of salt treatment, its expression in transgenic tobacco lines overexpressing *MzASMT 1* was also much higher than that in WT. The higher expression of *NtP5CS* may further lead to increased proline production in transgenic tobacco, which contributed to its salt tolerance. The DREB transcription factors, identified in a wide variety of plants, play important roles in plant stress response through the regulation of multiple stress response genes. There was a higher level of expression of *NtDREB* in transgenic lines compared with WT after salt treatment, which might further regulate the expression of other stress defensive genes, such as *NtERD10C*, *NtERD10D*, and so on. Overall, these results indicated that overexpression of *MzASMT 1* in transgenic tobacco lines enhanced salt tolerance by regulating the expression level of salt stress-related genes.

## Discussion

Melatonin, widely found in animals and plants, plays an important role in plants, especially when plants are subjected to a variety of abiotic stresses including drought, cold, salt, and extreme temperature (Lei et al., 2004; Tan et al., 2007; Wang et al., 2013). Extensive studies have revealed the crucial and indispensable roles that exogenous melatonin treatment play in increasing salt tolerance in diverse plant species (Campos et al., 2012; Liang et al., 2015; Jiang et al., 2016; Wang et al., 2016; Chen et al., 2018; Zeng et al., 2018). However, the effects of endogenous melatonin on the salt tolerance through genetic engineering have been reported much little. Recently, many reports indicated that the overexpression of various *COMT* in plants could increase melatonin production and enhance their salt tolerance (Liu D.D. et al., 2019; Sun et al., 2019; Zhang et al., 2019). However, few works have been conducted on the functions of *ASMT* in plant under salt stress. *MzASMT 1*, a drought-inducible gene, was cloned from *M. zumi* Mats, and the overexpression of which in *A. thaliana* enhanced melatonin production and improved their drought tolerance (Zuo et al., 2014). However, whether the overexpression *MzASMT 1* in tobacco could enhance their melatonin content and improve their salt tolerance has not been elucidated. In our results, the melatonin content in transgenic tobacco lines overexpressing *MzASMT 1* was significantly higher than that in WT (Figure 7), which was consistent with previous results in *A. thaliana*. The transgenic tobacco lines overexpressing *MzASMT 1* displayed better phenotypic morphology, such as much lower degrees of leaf wilting, much more fresh weight, higher plant height, and longer root under salt treatment, concomitant with higher RWC, chlorophyll content, and Fv/Fm value (Figures 2–4), indicating that transgenic lines had an enhanced salt tolerance compared with WT.

Reactive oxygen species are key signal molecules for plants, which benefit plant growth, especially when plants are subjected to environmental stresses. However, excessive ROS would damage membrane systems and negatively affect the normal growth of plants (Yang et al., 2017). Once the plant is under environmental stresses, the content of superoxide, hydrogen peroxide, and proline will increase. To alleviate or eliminate these oxidative stresses, its cells simultaneously initiate a series of response mechanisms and stress signals, such as the activation of cellular ROS scavenging mechanism, which can trigger the production of reactive oxygen scavenging enzymes and antioxidants, including POD and SOD which scavenge excessive ROS (Miller et al., 2010). In our results, there was no significant difference in H_2_O_2_ and O_2_^⋅–^ content between the transgenic lines and WT before salt treatment. Under salt treatment, the transgenic tobacco lines overexpressing *MzASMT 1* accumulated much lower H_2_O_2_ and O_2_^⋅–^ compared with WT, and the histochemical staining results are consistent with the above results (Figure 5). In accordance with the H_2_O_2_ and O_2_^⋅–^ content, there was a marked increase in POD, SOD, and CAT activities in transgenic lines compared with WT under salt treatment, and the corresponding gene expression level of *NtPOD*, *NtSOD*, and *NtCAT* is also consistent with the POD, SOD, and CAT activities (Figure 6).

Melatonin is a broad-spectrum antioxidant, and its primary function is to act as a free radical scavenger to protect plants from various environmental stresses, including salt stress. Different from the other conventional antioxidants, a single melatonin molecule can scavenge up to 10 ROS/RNS through the free radical scavenging cascade (Campos et al., 2012). Exogenous melatonin treatment significantly reduced salinity-induced ROS by melatonin or activating antioxidant enzymes, which has been confirmed in many plant species, such as soybean, *Malus hupehensis*, and kiwifruit (Campos et al., 2012). In cucumber, the activity of major protective antioxidant enzymes including SOD, CAT, POD, and APX in melatonin pre-treated plants was significantly higher than that in control plants (Wang et al., 2016). Under salt stress, exogenous melatonin application also significantly increased the activities of APX, CAT, SOD, POD, GR, and GPX in melatonin-treated seedlings compared to their non-treated counterparts (Jiang et al., 2016; Chen et al., 2018; Zeng et al., 2018). Sun et al. (2019) also indicated that the overexpression of *SlCOMT 1* alleviated antioxidant enzyme activity inhibition by salt stress, which could have been due to the accumulation of endogenous melatonin. Above all, endogenous or exogenous melatonin could increase the concentrations of antioxidants under stress conditions and improve stress tolerance (Wang et al., 2012; Cui et al., 2017; Li H. et al., 2017). In our results, the overexpression of *MzASMT 1* enhanced the content of melatonin in tobacco (Figure 7), and the *MzASMT 1* overexpression might also alleviate antioxidant enzyme activity inhibition by salt stress through the increase of melatonin content.

The accumulation of ROS will lead to serious oxidative damage and peroxidation of membrane lipids, which produces redundant MDA (Xie et al., 2008). The accumulation of MDA can cause damage to the cell membranes to some extent, which can reflect the degree of damage suffered by plant cells and the degree of membrane lipid damage. Therefore, the content of MDA can be used as a common indicator to judge the degree of stress experienced by the cells (Esfandiari et al., 2007). When plants subjected to various environmental stresses such as salt stress, drought, and low temperature, the content of MDA increased, whereas plants with a low MDA content might have a strong salt tolerance capacity (Xie et al., 2008; Zhang et al., 2019). In our results, the content of MDA was lower in the transgenic tobacco plants overexpressing *MzASMT 1* than that in WT under salt stress (Figure 8), indicating that the membrane system of the transgenic tobacco lines was slightly damaged, and the cell membrane of WT was seriously damaged. In addition, proline in small amount plays multiple roles, such as stabilization of membrane and proteins, redox homeostasis, and regulation of salt stress-responsive gene expression, which also plays important roles when plants encounter environmental stresses. In our results, all the transgenic plants had higher proline production and lower MDA content compared with WT (Figure 8), indicating that transgenic tobacco lines overexpressing *MzASMT 1* might enhance salt tolerance by inducing an increase in content of proline and a decrease in content of MDA.

It is well-known that stress-induced genes in plants are involved in the response to various environmental stresses and play important roles when plants encounter adverse environment (Li J. et al., 2017). Sun et al. (2019) indicated that the higher level of expression of stress-related genes in tomato plants might be one of the major mechanisms of improving the salt tolerance in tomato plants. Gao et al. (2018) also reported that melatonin-mediated induction of antioxidant responses might require the activation of ROS and MAPK. Actually, the three transgenic lines in our results had a higher level of expression of antioxidant-related genes (*NtPOD*, *NtSOD*, and *NtCAT*) in comparison with WT under salt stress, which was consistent with the greater activities of these antioxidant enzymes, indicating that antioxidant-related genes play important roles in melatonin-mediated salt tolerance. *NtERD10C* and *NtERD10D* encode late-embryogenesis abundant proteins of group 2, which partially bind water, stabilize labile enzymes, protect cellular and macromolecular structures, and reduce extensive membrane damage (Liu X. et al., 2009). In our study, three stress-responsive genes including *NtERD10C*, *NtERD10D*, and *NtLEA5* are induced in *MzASMT 1* transgenic tobacco lines under salt treatment, and the expression level of these three genes are much higher than that in WT (Figure 9). The Δ-pyrroline-5-carboxylate synthetase (P5CS), the rate-limiting enzyme in proline biosynthesis in plants, can control the level of proline in plants under both normal and stress conditions, which plays critical roles in improving the stress tolerance of plants (Hussain et al., 2000; Xu et al., 2011). There was a significant increase in the expression level of transgenic lines compared with WT under salt treatment, and the content of proline in transgenic lines is also much higher than that in WT under salt treatment (Figure 9), indicating that *P5CS* might play important roles in plants under salt treatment. The dehydration-responsive element-binding (DREB) transcription factors play important roles in regulating stress-related genes. Overexpressing *OsDREB2A* in soybeans enhanced salt tolerance by accumulating osmolytes, such as soluble sugars and free proline, and improving the expression levels of some stress-responsive transcription factors and key genes (Zhang et al., 2013). *VrDREB2A*, a DREB transcription factor from *Vigna radiata*, increased drought and high-salt tolerance in transgenic *A. thaliana* via transcriptional regulation of downstream genes containing the *cis-*element dehydration-responsive element (DRE) (Chen et al., 2016). The overexpression of *GmDREB2* activated the expression of downstream genes in transgenic *Arabidopsis*, resulting in enhanced tolerance to drought and high-salt stresses and did not cause growth retardation (Chen et al., 2007). A cotton (*Gossypium hirsutum*) DRE-binding transcription factor gene, *GhDREB*, confers enhanced tolerance to drought, high salt, and freezing stresses in transgenic wheat (Gao et al., 2009). In our results, the expression level of gene *NtDREB* in transgenic tobacco lines was much higher than that in WT under salt stress (Figure 9), indicating that *MzASMT 1* might improve plant salt tolerance by regulating the expression of stress marker genes.

**FIGURE 10:**
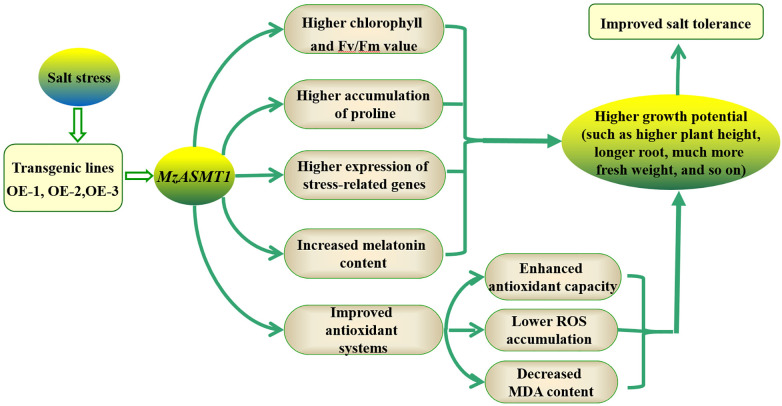
Proposed mechanisms of overexpression of *MzASMT 1* to enhance salt tolerance in tobacco.

Taken together, the present study focused on the functional roles of *MzASMT 1* in tobacco under salt treatment. The overexpression of *MzASMT 1* in tobacco increased the melatonin content compared with WT, and the transgenic lines overexpressing *MzASMT 1* also had higher accumulation of proline, lower accumulation of MDA concentration, and H_2_O_2_ and O_2_^⋅–^ content under improved antioxidant systems (including antioxidant enzyme system and corresponding genes), which contributed to their salt tolerance. The expression of the salt stress-related genes in transgenic lines overexpressing *MzASMT 1* was much higher than that in WT under salt treatment. Therefore, the transgenic lines overexpressing *MzASMT 1* exhibited lower degrees of leaf wilting; much more fresh weight; higher plant height; longer root; higher RWC of leaves, stem, and root; higher chlorophyll content; and Fv/Fm, indicating that the transgenic lines had higher salt tolerance capacity (Figure 10). The present study provided a theoretical basis for the application of *MzASMT 1* in improving plant salt tolerance, and the gene *MzASMT 1* may be a potential candidate gene in the functional exploration of salt tolerance mechanism in future studies.

## Data Availability Statement

The original contributions presented in the study are included in the article/Supplementary Material, further inquiries can be directed to the corresponding author.

## Author Contributions

WZ and ZW designed the experiments. TL, HW, and TW performed the experiments. FZ and XS assisted with the experimental procedures and data analysis. TL wrote the manuscript with the help of all authors. SQ provided supervision, funding, and reagents. All authors contributed to the article and approved the submitted version.

## Conflict of Interest

The authors declare that the research was conducted in the absence of any commercial or financial relationships that could be construed as a potential conflict of interest.

## Footnotes

1 https://www.ncbi.nlm.nih.gov/
