# Supplementary material for: Overexpression of MzASMT 1, a Gene From Malus zumi Mats, Enhances Salt Tolerance in Transgenic Tobacco
Source: Front Plant Sci. 2020 Oct 26;11:561903. doi: 10.3389/fpls.2020.561903 (PMC7649149; doi:10.3389/fpls.2020.561903)
Supplement: Supplementary file 1 [file Data_Sheet_1.docx]

**SUPPLEMENTARY MATERIALS**

Supplementary Table S1. Primers used in qRT-PCR analysis

| Primers | Sequence (from 5' to 3') |
| --- | --- |
| NtTubulin-F  NtTubulin-R  NtPOD-F  NtPOD-R  NtSOD-F  NtSOD-R  NtCAT-F  NtCAT-R  NtERD10C-F  NtERD10C-R  NtERD10D-F  NtERD10D-R  NtP5CS-F  NtP5CS-R  NtLEA5-F  NtLEA5-R  NtDERB3-F  NtDERB3-R  MzASMT1-F  MzASMT1-R  MzASMT1-RT-F  MzASMT1-RT-R | AGATGTTCCGTCGTGTCAGTG  TGCTTCCTCTTCATCCTCATATCC  CTCCATTTCCATGACTGCTTTG  GTTGGGTGGTGAGGTCTTT  ACCACCAGAAGCATCATCAGACT  TAATGTGACCTCCGCCGTTG  TTCTGCCCTTCTATTGTGGTTCC  ATGAGCACACTTTGGAGCATTAGC  AACGTGGAGGCTACAGATCG  GTTCCTCTTGGGCATGAGTT  GGCGGGCAAAAGAAGACAGA  GCCAAACAGCAGTAGCACAATCA  GACACGGACTGATGGAAGATTAG  GCACCTGAAGTCACCAGAATAA  GTTACCATACCACGTCCCATAG  GAGCTAGGACGCTCCATATTT  GCCGGAATACACAGGAGAAG  CCAATTTGGGAACACTGAGG  CATACCGGACATAATTGACCG  GACGGTGTGAGATCATAAGC  ATGGAGGGAGATGAAGCAAG  CTAAAGAAAAACTTCAATG |


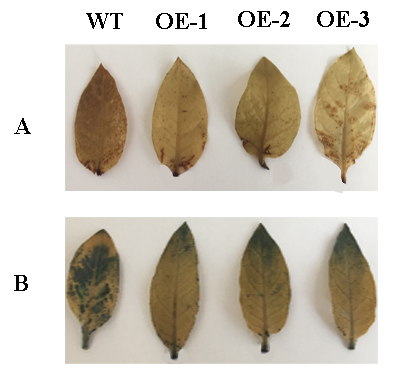
­

Supplementary Figure S1. Histochemical staining with DAB for detection of H_2_O_2_ (A) and with NBT for detection of O_2_.- (B) in transgenic lines and WT after 7 days of salt treatment and water treatment. (WT, wild-type; OE-1, Transgenic line OE-1; OE-2, Transgenic line OE-2; OE-3, Transgenic line OE-3).
